# Maize Thymidine Kinase Activity Is Present throughout Plant Development and Its Heterologous Expression Confers Tolerance to an Organellar DNA-Damaging Agent

**DOI:** 10.3390/plants9080930

**Published:** 2020-07-23

**Authors:** Manuela Nájera-Martínez, José Antonio Pedroza-García, Luis Jiro Suzuri-Hernández, Christelle Mazubert, Jeannine Drouin-Wahbi, Jorge Vázquez-Ramos, Cécile Raynaud, Javier Plasencia

**Affiliations:** 1 Departamento de Bioquímica, Facultad de Química, Universidad Nacional Autónoma de México, Ciudad de México 04510, Mexico; cronistachimalpa67@gmail.com (M.N.-M.); joped@psb.vib-ugent.be (J.A.P.-G.); jiro.suzuri@cienciaforense.facmed.unam.mx (L.J.S.-H.); jorman@unam.mx (J.V.-R.); 2 Institute of Plant Sciences Paris-Saclay (IPS2), CNRS, INRA, University Paris-Sud, University of Evry, Paris University, Sorbonne Paris-Cite, University of Paris-Saclay, Batiment 630, 91405 Orsay, France; christelle.mazubert@u-psud.fr (C.M.); jeannine.drouin-wahbi@u-psud.fr (J.D.-W.); cecile.raynaud@universite-paris-saclay.fr (C.R.); 3 Licenciatura en Ciencia Forense, Facultad de Medicina, Universidad Nacional Autónoma de México, Ciudad de México 04510, Mexico

**Keywords:** nucleotide metabolism, *Zea mays*, Arabidopsis, thymidine kinase, DNA damage

## Abstract

Thymidine kinase 1 (TK1) phosphorylates thymidine nucleosides to generate thymidine monophosphate. This reaction belongs to the pyrimidine salvage route that is phylogenetically conserved. In the model plant *Arabidopsis thaliana*, TK activity contributes to maintain nuclear and organellar genome integrity by providing deoxythymidine-triphosphate (dTTP) for DNA synthesis. Arabidopsis has two TK1 genes (*TK1a* and *TK1b*) and double mutants show an albino phenotype and develop poorly. In contrast, maize (*Zea mays L.*) has a single TK1 (*ZmTK1*) gene and mutant plants are albino and display reduced genome copy number in chloroplasts. We studied the role of *ZmTK1* during development and genotoxic stress response by assessing its activity at different developmental stages and by complementing Arabidopsis *tk1* mutants. We found that *ZmTK1* transcripts and activity are present during germination and throughout maize development. We show that ZmTK1 translocation to chloroplasts depends on a 72-amino-acid N-signal and its plastid localization is consistent with its ability to complement Arabidopsis *tk1b* mutants which are hypersensitive to ciprofloxacin (CIP), a genotoxic agent to organellar DNA. Also, *ZmTK1* partly complemented the Arabidopsis double mutant plants during development. Our results contribute to the understanding of TK1 function in monocot species as an organellar enzyme for genome replication and repair.

## 1. Introduction

Pyrimidine nucleotides are essential components of DNA and RNA. These molecules also participate in bioenergetic processes as well as in the synthesis of sucrose, polysaccharides, and phospho- and glycolipids. In plants, they are also involved in the biosynthesis of several secondary metabolites [1]. De novo pyrimidine nucleotide biosynthesis starts by the action of the carbamoyl phosphate synthetase that uses carbonate, ATP and the amino group from glutamine to generate carbamoyl phosphate. Biosynthesis proceeds in plastids and mitochondria to yield CTP. Ribonucleotide reductase reduces the -OH at the ribose C2’position in UDP to produce dUDP which serves as substrate for a nucleoside diphosphate kinase and is then dephosphorylated by a pyrophosphatase to yield dUMP. This intermediate is methylated by thymidilate synthase to generate 5’-deoxythymidine monophosphate (dTMP) that is sequentially phosphorylated to form dTTP [2,3,4]. 

Nucleotides and deoxynucleotides are also produced by the salvage route that reutilizes pyrimidine bases and nucleosides derived from preformed nucleotides. Among the pyrimidine bases, only uracil is recycled by the action of a phosphoribosyltransferase, whereas the pyrimidine nucleosides, uridine, cytidine and deoxycytidine are salvaged through phosphorylation to generate the monophosphorylated nucleotides [2].

In a similar way, thymidine is salvaged through its phosphorylation by thymidine kinase that uses ATP to generate dTMP. Moreover, thymidine can also be phosphorylated by non-specific phosphotransferases (NSPT) that use AMP as a phosphate donor. The study of the role of salvage biosynthesis in plant physiology has been limited to a few models that represent distinct stages of plant growth such as embryo formation and germination, shoot organogenesis, storage organs development and sprouting, and leaf senescence [5]. Most of our knowledge comes from labeling studies with pyrimidine bases and nucleosides to follow their incorporation onto nucleic acids, or degradation to yield CO_2_ and NH_3_; from such studies, it was established that the salvage pathway operates in both angiosperms and gymnosperms [2].

During somatic carrot embryogenesis the pyrimidine salvage pathway operates for the initiation and maturation of the embryos, specially through NSPT activity. Likewise, during white spruce embryo maturation NSPT activity is higher than that of TK, but the latter increases gradually during germination, and after 6 days, it is 4-fold that of NSPT. During shoot formation in tobacco callus, thymidine utilization increases sharply in shoot-forming tissue, but TK activity fluctuates during the culture period and is 2- to 3-fold lower than that of the NSPT [3]. These studies have showed the differential role of TK to synthesis of nucleotides of thymidine in distinct developmental stages.

In plants, an ATP-dependent activity of thymidine phosphorylation was detected during germination of wheat [6] and maize [7] seeds. Also, a few TK genes have been described in plant species; in rice, TK mRNA levels are higher in mature as well as in meristematic tissues, thus the pyrimidine salvage pathway might function in other events requiring DNA synthesis, such as DNA repair. The *Arabidopsis thaliana* genome contains two TK genes (*TK1a* and *TK1b*), and single homozygous mutants of any of these genes show a lower growth rate at seedling developmental stages and TK activity is required for optimal growth as the double mutant displays an albino phenotype and delayed development [8]. *TK1a* and *TK1b* proteins have distinct subcellular localization; while the former is a cytosolic enzyme, the latter localizes in the mitochondria [9] and chloroplasts [8]. *TK1a* and *TK1b* show ubiquitous expression during plant growth and development [9,10]. TK1 activity has a substantial role during early plant growth for plastid development and maintenance before germinating seedlings turn autotrophic [8]. *TK1* genes in Arabidopsis display a differential response to ultraviolet-C (UV-C) radiation, while *TK1a* gene is strongly induced 1–3 h post irradiation, *TK1b* transcript levels remain constant. Moreover, overexpression of the *TK1a* gene confers tolerance to genotoxic stress [10]. These differences are also reflected in their roles in genotoxic stress response: whereas *TK1a* contributes to DNA repair of the nuclear genome, *TK1b* is involved in maintaining the integrity of organellar genomes [8]. 

In contrast to Arabidopsis, maize (*Zea mays L.*) contains a single *ZmTK1* gene (*CPTK1*) in its genome. The enzyme is localized in both mitochondria and chloroplasts, and it is essential for plastidial genome replication, as maize mutants show an albino phenotype and reduced chloroplast DNA copy numbers, these deficiencies leading to early death [11]. In this work, we further explored the role of maize TK1 during development and genotoxic stress response by studying its activity at different developmental stages and by complementing Arabidopsis *tk1* mutants. Furthermore, we determined that the *N*-terminal signal sequence in ZmTK1 is required for chloroplast translocation.

## 2. Results

### 2.1. *ZmTK1* Transcript and Activity Are Present throughout Maize Development

**Figure 1:**
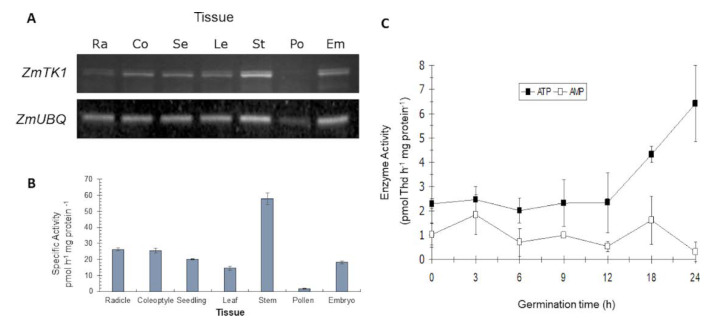
*ZmTK1* transcript and enzymatic activity are ubiquitously present during maize development. (**A**) Transcript levels of *ZmTK1* in radicles of 2 days seedlings (Ra), coleoptiles of 5-day seedlings (Co), 10-day-old seedlings (Se), leaf (Le) and stem (St) of 20-day-old plants, dehiscent pollen (Po) and mature embryos (Em). Ubiquitin transcript used as a loading control. (**B**). Thymidine kinase enzymatic activity in the same maize tissues. (**C**) TK activity and non-specific phosphotransferase (NSPT) activity during maize germination. For Figure 1B,C, values represent average ± standard deviation of three technical repeats from three independent protein extracts replicates.

Since both *TK1* Arabidopsis genes have a ubiquitous expression throughout plant development [10], we tested whether this was also true in maize. To address this, we first analyzed transcript levels by semi-quantitative reverse transcription polymerase chain reaction (RT-PCR), using total RNA from various tissues representing different developmental stages in maize (radicles from 2-day seedlings, coleoptiles from 5-day seedlings, 10-day-old seedlings, leaf and stem from 20-day-old plants, dehiscent pollen and mature embryos). Figure 1A shows that *ZmTK1* was expressed in all tissues, although transcript levels varied among them. For some tissues, a double band was clear however, upon sequencing the amplicon we did not found evidence of alternate splicing of the mRNA. To associate mRNA levels with the protein function, we measured thymidine kinase enzyme activity in soluble protein extracts of these tissues. We detected TK1 activity in all tissues tested but significant differences were observed among them; stems harbored the highest values, whereas pollen showed the lowest TK1 activity (Figure 1B). Thus, our results reveal that *ZmTK1* is expressed throughout the plant to yield an active enzyme. 

### 2.2. *ZmTK1* Activity Is Associated with DNA Synthesis during Germination

**Figure 2:**
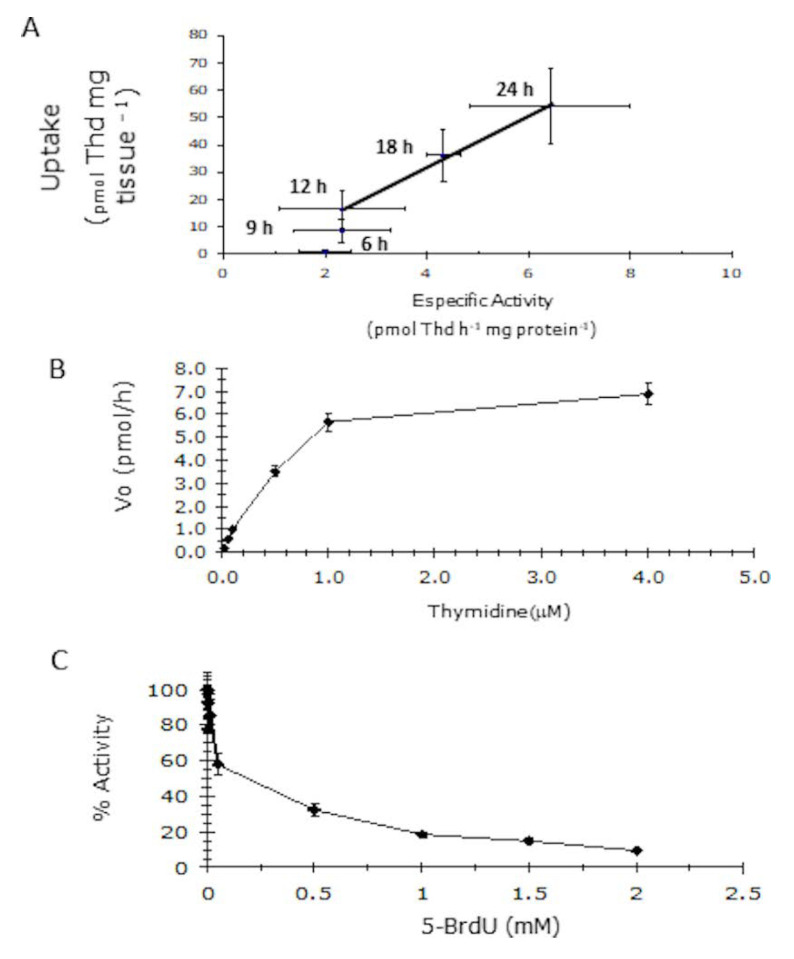
Biochemical characterization of maize thymidine kinase activity. (**A**) Association between TK enzymatic activity with DNA synthesis, assessed by ^3^H-thymidine uptake, in maize embryo axes. (**B**) Michaelis–Menten plot of the TK activity for increasing thymidine concentrations. (**C**) Sensitivity of maize thymidine kinase activity to 5-bromo deoxyuridine. Values represent average ± standard deviation of three technical repeats from three independent protein extracts replicates.

In Arabidopsis, thymidine kinase activity is required for proper seedling establishment [8], thus we evaluate it during maize germination to gain more insight on its role at this developmental stage. Because thymidine phosphorylation can also be carried out by non-specific phosphotransferases, both TK and NSPT enzymatic activities were determined during embryo axes germination using ATP and AMP as phosphate donor, respectively. Low enzymatic levels were detected during early times in germination, but after 12 h imbibition, TK activity increased while NSPT activity remained constant and by 24 h, thymidine phosphorylation activity was 10-fold higher for TK than NSPT (Figure 1C). These results point out that TK activity might contribute to fill the dNTP pools for DNA synthesis. To gain insight into this, [^3^H]-Thd uptake into DNA was correlated with TK enzyme activity in the apex of maize embryo axes during germination. Low levels of Thd uptake and TK activity were detected at early germination times (3 h–6 h) but after 12 h imbibition, a significant rise in Thd uptake was recorded and increased gradually, as already reported [12]. Such increment in Thd uptake was accompanied by a significant rise in TK enzymatic activity (Figure 2A).

Unlike deoxyribonucleoside kinases which have broad specificity towards deoxyadenosine, deoxyguanosine and deoxythymidine, TKs only phosphorylate deoxythymidine and deoxyuracil [13]. Because TK enzymatic activity was enriched in 24-h germinating embryos, we were able to characterize it biochemically by determining its kinetic parameters and sensitivity to bromo-deoxyuridine, a thymidine analog, that competitively inhibits TK1 activity. Under our experimental conditions, the apparent Km value for thymidine was 1.2 ± 0.3 µM (Figure 2B) and enzyme activity was strongly inhibited by 5-BrdU (Figure 2C).

### 2.3. *ZmTK1* Is Directed to Organelles through an Amino Leader Peptide

Phylogenetic analyses on plant TKs shows that a single gene is present in non-flowering plants and in Poaceae, such as maize [11]. In contrast, eudicots acquired a second gene, and if Arabidopsis faithfully represents most of dicot plants, TK1b-type proteins are targeted to organelles and would be expected to have the same functions as the organellar TK1 from Poaceae [11]. However, all the domains of TK1 protein are conserved between plants and other eukaryotes. 

As reported before, Arabidopsis expresses two *TK1* paralogs with distinct subcellular localization; while AtTK1a resides in the cytosol, AtTK1b is directed to chloroplasts and mitochondria [8]. Although ZmTK1 shares a higher identity to AtTK1a (64%) than to AtTK1b (60%), it contains an extension at the N-terminus comparable to that of AtTK1b (Supplementary Materials Figure S1) and is targeted to both plastids and mitochondria [11]. Initial evidence in monocots pointed to an organellar location for plant TK1, as chloroplasts isolated from rye seedling leaves contain 5- to 10-fold higher thymidine phosphorylating activity than the cytosolic fraction [14].

**Figure 3:**
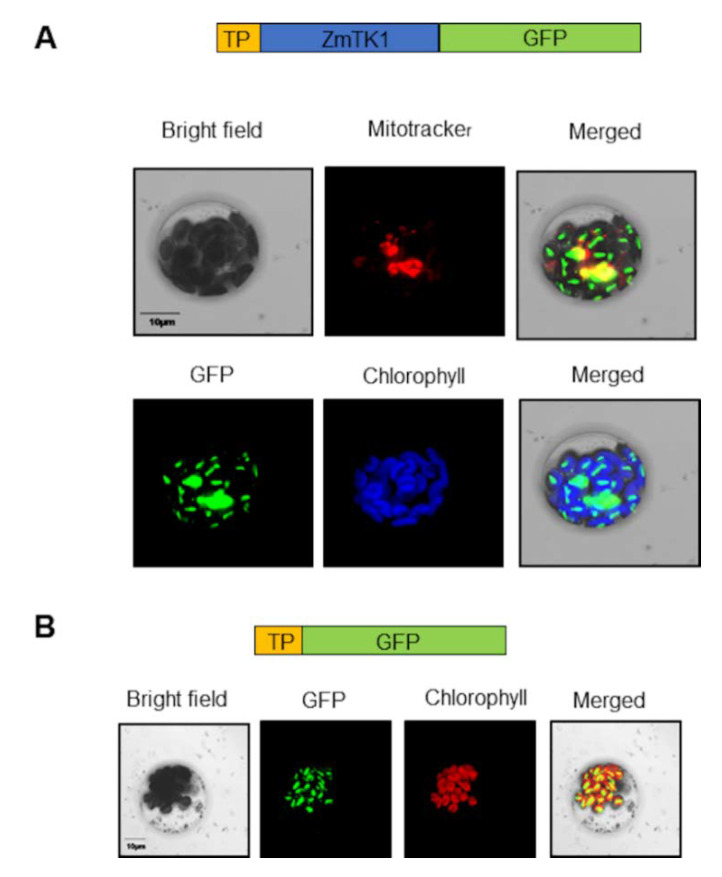
Transient expression of 35S::ZmTK1-GFP in Arabidopsis protoplasts. (**A**) Colocalization of green fluorescent protein (GFP) with mitotracker and chlorophyll fluorescence. (**B**) A 72-aminoacid sequence at the N-terminus of ZmTK1 (35S::TP-ZmTK1-GFP), drove GFP to the chloroplast. The bar represents 10 µm.

To determine whether this N-leader sequence is functional, we used a transient expression assay in Arabidopsis protoplasts. The full length ZmTK1 protein was localized in chloroplasts as chlorophyll auto-fluorescence clearly colocalized with a green fluorescent protein (GFP) signal (Figure 3A). We also found that 35S::ZmTK1-GFP colocalized with mitotracker, a mitochondrial marker (Figure 3A) consistent with previous reports [11]. 

To examine if organelle localization was dependent on the N-terminus leader sequence, we generated a chimeric construction containing the first 72 amino-acids from the TK1b fused to GFP and transiently expressed it in Arabidopsis protoplasts. We defined a 72-aminoacid sequence predicted by TargetP (http://www.cbs.dtu.dk/services/TargetP/ at Kongens Lyngby, Denmark) that is rich (16%) in Ser, lacks Tyr and contains only one acidic amino-acid (Glu) (Supplementary Materials Figure S1). Such amino-acid composition, among other properties, is compatible with a role of the sequence as an organellar localization signal [15,16].

The 72 amino-acid signal sequence was sufficient to direct translocation of GFP to chloroplast (Figure 3B). A third GFP chimeric fusion (35S::ZmTK1Δ84-GFP) was tested that lacked the N-signal sequence plus 12 amino-acids from the mature protein, including Gly and Pro belonging to the ATP binding domain. This chimeric protein was translocated neither to chloroplasts nor to mitochondria and appeared to form aggregates in the cytosol (Supplementary Materials Figure S2). The N-terminus of ZmTK is thus necessary and sufficient for chloroplast and mitochondria targeting.

### 2.4. *ZmTK1* Complemented *TK1* Deficiency in Arabidopsis Mutants

**Figure 4:**
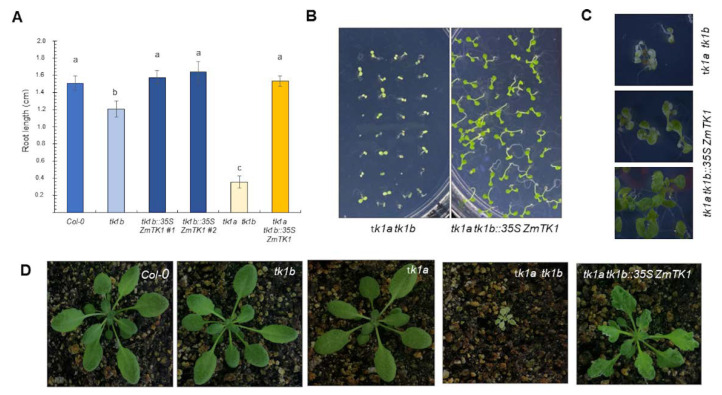
*ZmTK1* complements Arabidopsis TK1 double mutants. (**A**) Root length of Col-0, Arabidopsis TK mutants and complemented mutants. Seedlings were germinated on Gamborg´s B5 (GB5) agar medium and root length was measured in 9-day seedlings; values are average ± standard deviation (SD, *n* = 20). Different letters indicate significant mean differences as analyzed by analysis of variance (ANOVA) followed by Tukey’s comparison test (*p* < 0.01). Representative phenotypes of *tk1a tk1b* Arabidopsis mutants and complemented with *ZmTK1* during seedling development (**B**,**C**) and in adult plants (**D**).

In Arabidopsis, *tk1b* mutant shows a growth deficiency phenotype reflected by shorter roots and smaller cotyledons when seeds are germinated without an exogenous carbon source. Likewise, double mutant plants are viable but develop poorly at the seedling stage with short roots and albino cotyledons [8,13]. To test whether *ZmTK1* is able to complement the deficiency of organellar TK1b in Arabidopsis, we expressed the *ZmTK1* cDNA in the single *tk1b* mutant and in the double mutant background (*tk1a tk1b*). Sesquimutant Arabidopsis plants (*tk1a−/+tk1b−/−*) were transformed with the 35S::ZmTK1-GFP construct. In the T1 segregation, single, sesqui-, and double-mutant plants were recovered and genotyped. *ZmTK1* expression complemented *TK1* deficiency in the *tk1b* mutant and in the double-mutants during seedling development as root length values were not significantly different of those from the wild-type genotype (Figure 4A,B). However, cotyledons still showed the chlorotic phenotype (Figure 4C) and plants developed, albeit smaller than the wild-type or single mutant plants and with lobulated leaves (Figure 4D). Complementation allowed survival and development, and plants were fertile.

In [9], the authors showed that the rice TK gene (*OsTK*; LOC_Os03g02200) complements the Arabidopsis double *TK1* mutant. The rice protein, like most monocot TKs, harbors the N-terminal transit peptide that likely drives the enzyme to both organelles. Complementation rescued the albino phenotype, but plants still developed variegated cotyledons, and were smaller than the Col-0 wild type. However, the lobulated leaf phenotype that we report for the complementation with the maize cDNA was not observed. These results suggest that ZmTK1 was expressed at insufficient levels, or with an inappropriate expression pattern, due to the use of the 35S promoter. Another possibility is that the heterologous peptide signal was unable to drive the enzyme to chloroplast and mitochondria at adequate levels. Likewise, the HsTK1, a cytosolic enzyme which lacks the N-signal peptide also recovers the albino phenotype in the TK1 double mutant, but plants are also smaller than the wild-type Col-0 [9].

### 2.5. *ZmTK1* Contributes to Organellar Genome Integrity in Response to Genotoxic Agents

In Arabidopsis, the *TK1a* gene is up-regulated by UV-C radiation, and zeocin, a drug that induces double-strand breaks in the nuclear genome. Single *tk1a* mutants are hypersensitive to zeocin, whereas its overexpression confers tolerance to genotoxic agents [8,10]. In contrast, the *tk1b* single mutant displays hypersensitivity to compounds that generate DNA damage specifically in organelles as ciprofloxacin (CIP) and novobiocin. ZmTK1 has an organellar location and is required for the efficient replication of the plastid genome in maize [11]. To evaluate its function in response to CIP, a genotoxin that induce double-strand breaks in the organellar genomes, sensitivity assays to this antibiotic were performed in Arabidopsis mutants complemented with 35S::ZmTK1-GFP.

**Figure 5:**
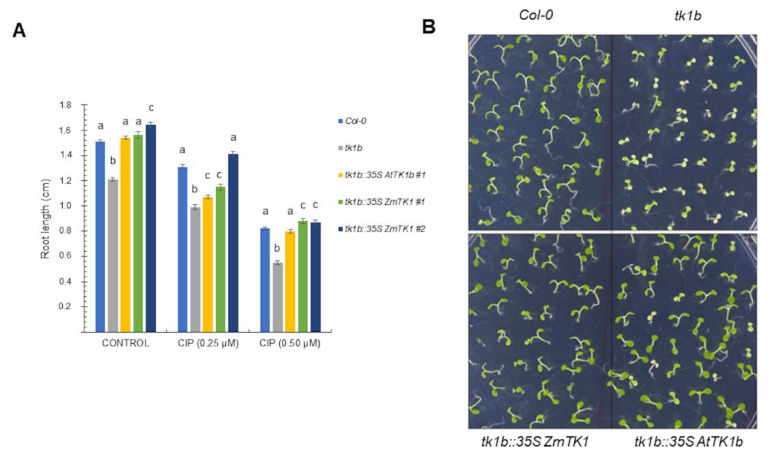
Expression of ZmTK1 in *tk1b* mutant confers tolerance to organellar genotoxin. Col-0, *tk1b* mutant, and *tk1b* mutants complemented with the native gene (*AtTK1b*) or the maize gene (*ZmTK1*) were germinated on GB5 agar medium supplemented with ciprofloxacin (CIP) (**A**) and root length was measured in 9-day-old seedlings. Values are averages ± SEs (*n* = 20) are shown, and different letters in each treatment indicate significant mean differences, as analyzed by ANOVA followed by Tukey’s comparison test (*p* < 0.01). (**B**) Representative phenotypes of Col-0, *tk1b* mutants and complemented mutants grown in presence of 0.5 µM CIP.

In Arabidopsis, *tk1b* mutants are hypersensitive to CIP [8]. As we observed with the double mutants, *ZmTK1* efficiently complemented the mutation and transgenic lines growth was comparable to that of wild-type seedlings in GB5 media lacking sucrose, as determined by root length (Figure 5A). Moreover, when these seedlings were challenged with CIP (Figure 5A,B) their response, determined by seedling root length, was comparable to that of wild type plantlets and of those of *tk1b* mutants complemented with the native gene. Mutants on *tk1b* showed chlorotic cotyledons and a shorter root when exposed to 0.5 µM CIP, but complementation with the native gene or the maize TK1 gene rescued the phenotype (Figure 5B).

**Figure 6:**
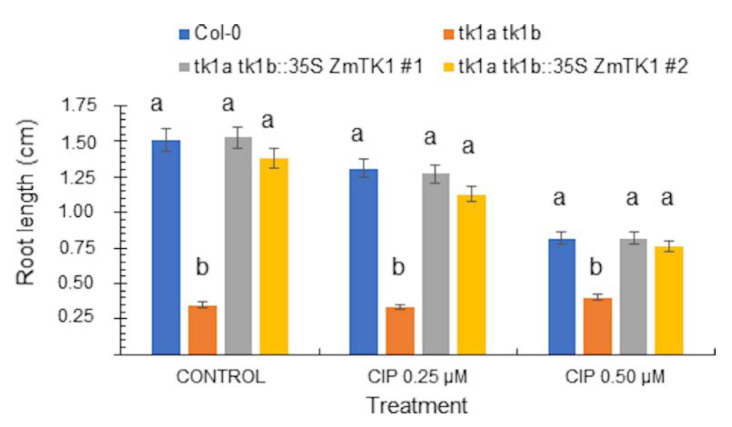
Expression of *ZmTK1* in Arabidopsis TK1 double mutant allows the plants to develop in presence of CIP. Col-0, *tk1a tk1b* double mutant, and double mutants complemented with *ZmTK1* were germinated on GB5 agar medium supplemented with CIP and root length was measured in 9-day-old seedlings. Values are averages ± SEs (*n* = 20) are shown, and different letters in each treatment indicate significant mean differences, as analyzed by ANOVA followed by Tukey’s comparison test (*p* < 0.01).

Moreover, *ZmTK1* overexpression in the double (*tk1a tk1b*) mutant allowed them to grow in presence of CI P < with a comparable response to that of the wild-type Col-0 (Figure 6). These results suggest an interchangeable function between the cytoplasmic and organellar paralogs. To further explore this, we expressed the Arabidopsis *TK1a* gene in the *tk1b* background and found that it partly complemented as root growth is not fully restored, tested in the absence and presence of CIP (Supplementary Materials Figure S3). Our results point out to the importance of the subcellular localization of AtTK1b and ZmTK1 activity for providing dTTP employed for organellar DNA synthesis.

## 3. Discussion

### 3.1. *ZmTK1* Activity Is Detected at the Onset of Germination and throughout Plant Development

*ZmTK1* transcript and activity were detected throughout plant development, these results are consistent with the expression reported for Arabidopsis ortholog genes, in which both *TK1* genes are ubiquitously expressed [9,10]. The presence in differentiated tissues such as stems and leaves of 20-day old plants is consistent with its role for plastid genome replication [11]. In contrast to Arabidopsis *TK1* genes, which showed high expression levels of both genes in reproductive tissues such as anthers and pollen [9,10], low abundance of transcripts and TK1 activity were detected in maize pollen. It could be significant as pyrimidine nucleotides should be required for pollen tube growth [17], suggesting that the de novo pathway has a relevant role in this maize tissue. Likewise, ZmTK1 activity was detected in proliferating tissues such as embryo and radicle, suggesting that the synthesis of nucleotides is required for the replication and repair of organellar DNA in actively dividing cells. Salvage pathways are particularly important during seed and embryo germination as they transform the stored free bases and nucleosides into nucleotides before the onset of the de novo nucleotide synthesis [2,3]. DNA repair synthesis occurs at early stages during seed imbibition with the first burst of metabolic activity [18,19]. Both repair of nuclear DNA and replication of mitochondrial DNA are critical during the imbibition of leek embryo [20]. Loss of vigour has been associated with reduced levels of nucleosides and nucleotides needed for nucleic acid synthesis and repair during cell expansion and division in the embryo of the germinating wheat seed [21]. In maize embryos, extensive DNA repair is required during the first hours of germination as single-strand breaks on DNA occur in the meristematic cells of the dry embryo, and DNA synthesis is very active for organellar genome replication and DNA repair [22]. Also, active DNA synthesis can be detected in germinating maize embryo axes, reaching its highest values between 24–40 h, which is associated to the S-phase of the first cell cycle [12,23].

Here, we found that [^3^H]-Thd incorporation was low between 3 h and 9 h germination, and a sharp increase after 12 h, reaching a peak at 24 h imbibition. The ZmTK1 activity pattern was similar to that of [^3^H]-Thd incorporation, as it increased notably between 12 h and 18 h imbibition, probably contributing to provide dNTPs mainly for organelle DNA synthesis and repair [23], although its involvement during synthesis of nucleotides for the S phase of the first cell cycle cannot be ruled out.

Our results agree with those obtained on other monocots; in wheat embryos, DNA synthesis increases at 12 h after water imbibition [6] as well as in maize [12,24] with a concomitant rise in TK activity [7]. We found that during germination of maize the NSPT activity also can be detected but did not vary during the imbibition as TK1 activity, thus highlighting the role of TK1 in providing dTPP for DNA synthesis at these early stages. We confirmed that enzymatic activity detected in our studies were specific from TK1, using 5-bromo-desoxy-uridine (BrdU), which is a specific inhibitor for TK1. The decrease of TK1 activity was dependent of the BrdU concentration added to the reaction. Furthermore, the high sensitivity and specificity of the assay allowed us to determine some kinetic parameters for the reaction, we found that the apparent K_M_ value for deoxythymidine was 1.2 ± 0.3 µM, which is comparable to those of the recombinant Arabidopsis enzymes. K_M_ values for deoxythymidine were 0.62 µM and 1.4 µM for AtTK1a and AtTK1b, respectively [13].

### 3.2. Role of *ZmTK1* in the Organelle DNA Replication and Repair

A previous report demonstrated that *ZmTK1* is located in both plastids and mitochondria, but its deficiency affects mostly replication of the plastid genome [11]. Here, we confirmed the subcellular localization of *ZmTK1* in both organelles that is dependent on an N-terminal signal peptide. Similarly, to the dependence of TK1b from Arabidopsis to this signal peptide for the mitochondria location [9].

In Arabidopsis, the *tk1a tk1b* double mutant development is severely affected and shows an albino phenotype. The relevant function of TK1 activity during plant development is due to its role for plastid genome maintenance in germinating seedlings upon reaching their autotrophic stage [8]. These phenotypes are similar to those of mutants on genes for proteins of other salvage pathways involved in the biogenesis of chloroplast [25,26]. We showed that *ZmTK1* was able to complement partly the developmental defects of the Arabidopsis *tk1a tk1b* double mutants and to restore tolerance of *Attk1b* mutants to compounds that damage to organellar DNA.

Both, TK1a and TK1b, have similar kinetic parameters [13], but their subcellular localization suggest distinct roles; while TK1a is particularly required for nuclear genome repair, TK1b appears to be involved exclusively in the safeguarding of organellar genomes [8]. Their distinct subcellular localization indeed confers them different functions, since the Arabidopsis proteins are not fully redundant during the transition for heterotrophy to autotrophy [8]. Thus, our results are consistent with these observations, in the double *TK1* mutants, the albino phenotype recedes but variegation was still present on the cotyledons in seedlings and smaller size and lobular leaves in adult plants. The fact that an organellar enzyme could partially complement a cytoplasmic function or vice versa might be due to the active transport between these cellular compartments. For instance, in Arabidopsis at least 49 transport proteins have been identified that keep the homeostasis of the nucleotide pool [27].

Here, our results revealed that *ZmTK1* can complement the lack of Arabidopsis *TK1b*, supporting its contribution to the maintenance of the nucleotide pool for organellar DNA replication and repair. The presence of a single *TK1* gene in maize suggests that the thymidine salvage pathway is located specifically in organelles and not in the cytosol. The de novo pathway thus likely provides the nucleotides required for the replication and repair of the nuclear genome. Consistent with this hypothesis, the *ZmRNR1* and *ZmTSO1* that are involved in de novo nucleotide biosynthesis genes are up-regulated in response to camptothecin (an inhibitor of topoisomerases used to induce DNA stress specifically in the nucleus) in immature embryo of maize [28], but expression of *ZmTK1* did not vary under this treatment. Whether this compartmentalization of functions is conserved in other monocot plants that contain a single *TK1* gene remains to be established. Comparative studies between monocot and eudicot plants will allow a better understanding on the plant nucleotide metabolism and its role on development and stress responses.

## 4. Materials and Methods

### 4.1. Biological Materials

Mature seeds of the landrace “Chalqueño” were employed for all experiments. Maize seeds were surface disinfected with a 0.5% sodium hypochlorite solution and rinsed with sterile water. Seeds were germinated in 1% agar at 27 °C and radicles and coleoptiles were collected at the indicated times. Otherwise, germinated seeds were transferred to soil to obtain plant tissue. Other tissues used were the plant leaf 3 of 14 and 24 days of age and the stem of the latter. Embryo axes were manually dissected from dry seeds. For germination experiments, the dissected embryo axes were surface disinfected as described above and imbibed in Murashige and Skoog (MS) agar at 27 °C. Samples were taken at different times and the apical sections of the germinated embryo axes (2-to-5 mm) were dissected for protein extraction.

*Arabidopsis thaliana* seeds were surface-sterilized by treatment with a solution containing 5% sodium hypochlorite for 20 min, washed and imbibed in sterile-water for 2–4 days at 4 °C to obtain homogeneous germination. Seeds were sown on commercially available GB5 agar medium (Gamborg´s B5 Basal Medium Sigma-Aldrich Co., St. Louis, MO, USA), solidified with 1% agar (Phyto-Agar HP696, Kalys) and grown in a long-day (16 h light, 8 h night, 21 °C) growth chamber. For selection of transgenic lines, seeds of the T1 generation were sown on sand and watered with a solution of glufosinate (7.5 mg/L) or in GB5 supplemented with hygromycin 30 µg/mL according to the resistance. Independent lines were self-fertilized, and homozygous lines of the T3 generation were used for all subsequent experiments.

*Arabidopsis thaliana* ecotype Columbia (Col-0) was used as the wild-type plant throughout this study. *Arabidopsis thaliana* ecotype Columbia (Col-0) and the T-DNA insertion mutant lines [SALK_097767 (*tk1a-2*); SALK 074256 (*tk1b*)] were obtained from the Arabidopsis Biological Resource Center at the Ohio State University (https://abrc.osu.edu). Crosses were performed between heterozygous *tk1a-2* mutant and homozygous *tk1b* mutant to obtain double mutants as was described in [8].

### 4.2. Genotoxic Treatments

To evaluate the sensitivity to genotoxic agent (CIP; Sigma-Aldrich, St. Louis, MO, USA), disinfected *Arabidopsis* seeds were sown in GB-5 agar medium supplemented with the genotoxin, CIP at 0.25 µM and 0.50 µM. Seeds were stratified under dark at 4 °C for 48 h and then germinated in a growth chamber at 22 °C under long-day photoperiod (16 h light; 8 h dark). After 9 days, plantlets were analyzed for root length.

### 4.3. Cloning and Plasmid Constructs

*ZmTK* cDNA was cloned from total RNA extracted from the coleoptiles of 6-day old seedlings- RT-PCR amplification was performed with primers that align in 5′-UTR and 3′-UTR sequences (Supplementary Materials Table S1), using the high fidelity polymerase Advantage HD (Clontech, Mountain View, CA, USA). The cDNA was cloned in pGEM^®^-T Easy vector (Promega, Madison, WI, USA) and ligation products were used to transform competent *E. coli* DH5α. The full cDNA sequence was obtained and registered in Gen-Bank (Accession Number AM492793).

### 4.4. *ZmTK1*–GFP Fusion Constructs

Gateway^®^ vectors were employed to generate translational fusions of *ZmTK* and green fluorescent protein (GFP), using the primers listed in Supplementary Materials Table S1 and Advantage HD Polymerase (Clontech, Mountain View, CA, USA). The amplified PCR products were cloned into the entry vector, pDONR™221 (Thermo Fisher Scientific, Carlsbad, CA, USA), using BP clonase reaction (Invitrogen, Carlsbad, CA, USA). Then, these entry clones were digested with the restriction enzyme *MluI* (FD0564, Thermo Scientific, Carlsbad, CA, USA) and DNA fragments were subcloned into the pEarleyGate 103 binary vector to generate GFP fusions driven by the CaMV 35S promoter [29] by LR clonase reaction (Invitrogen). These constructs were used for complementation of Arabidopsis mutants and to determine subcellular location. Three constructs were generated: a full-length ZmTK fused to GFP (hereafter referred to as 35S::ZmTK-GFP), a 72-aminoacid N-terminal sequence fused to GFP (35S::TpZmTK-GFP) and a 195-aminoacid C-terminal sequence fused to GFP (35S::ZmTKΔ84). The latter fusion lacked the N-terminal signal sequence and 12 amino-acids from the mature protein, including a region from the ATP-binding domain.

### 4.5. *ZmTK1* Complementation of Arabidopsis TK1 Mutants

Arabidopsis *tk1a2 tk1b+/−* sesquimutants [8] were transformed with a plasmid encompassing the 35S::ZmTK-GFP construct. After selection with glufosinate as described above, complemented *tk1a tk1b* double mutants were identified by PCR genotyping of the T2 generation, and all subsequent experiments were performed on plants of the T3 generation selected for homozygosity for the 35S::ZmTKGFP construct.

### 4.6. RNA Extraction and Reverse Transcription Polymerase Chain Reaction (RT-PCR)

Total RNA was extracted from maize tissues (2–3 g) using the Trizol reagent (Invitrogen, Carlsbad, CA, USA) according to the manufacturer instructions followed by precipitation with isopropanol. Reverse transcription was performed with 1 µg of RNA using ImProm-II reverse transcriptase (Promega, Madison, WI, USA) in presence of 61 µM oligo dT-VN.

Specific oligonucleotides for the synthesis of *ZmTK1* cDNA were designed (Supplementary Materials Table S1) that amplified a 309-bp fragment from the 3’-UTR and a 711-bp amplicon from maize ubiquitin gene (*ZmUBQ*) was employed as loading control [30]. PCR amplification was carried out in an Applied Biosystems GeneAmp System 9700 thermocycler (Applied Biosystems, Foster City, CA, USA) programmed for initial denaturation 94 °C for 5 min, denaturation at 94 °C for 1 min, annealing at 55 °C (*ZmTK1*), 58 °C (*ZmUBQ*) for 1 min, extension at 72 °C for 1 min, 32 cycles for *ZmTK1*, 30 cycles for *ZmUBQ*, terminal extension at 72 °C for 5 min.

### 4.7. Protein Extraction and Analysis

Proteins were extracted from the various maize tissues by freezing them in liquid nitrogen in 1.5 mL of protein extraction buffer (70 mM Tris pH 7.5, 1 mM MgCl_2_, 25 mM KCl, 5 mM Na_2_EDTA 2H_2_O pH 8.0, 0.25 mM sucrose, 15 mM β-mercaptoethanol, 0.1%Triton X-100, protease inhibitor cocktail Complete Mini, Roche). The homogenized tissue was kept on ice all the time and centrifuged at 16,200× *g* for 30 min to obtain the cytosolic soluble fraction. Total protein was quantified according to Bradford (1976) using the Bio-Rad Protein Assay reagent (Bio-Rad Laboratories, Inc., Hercules, CA, USA).

### 4.8. Thymidine Kinase Activity

Thymidine kinase enzymatic activity was determined by adapting the protocol reported by [31]. Soluble protein (500 µg) extracted from maize tissues was incubated with an isotopic mixture of thymidine and [methyl-^3^H]-thymidine (48 Ci/mmol) in reaction buffer (100 mM Tris, 10 mM MgCl_2_, 10 mM NaF, 10 mM, ATP, 2 mM β-mercaptoethanol) for 2 h at 37 °C. At the end of the incubation period, 0.1 mL of the reaction mixture was mixed in an Eppendorf tube with 2 mL of precipitation solution (100 mM LaCl_3_; 5 mM triethanolamine) and centrifuged for 10 min at 200× *g* in a Jouan MR1814 centrifuge. The precipitate was washed once with the precipitation solution and dissolved in 300 µL of 0.05 N HCl and 3 mL of Bray scintillation liquid. Total cpm were recorded in a Beckman LS 6000IC scintillation counter. For enzyme kinetic analysis, data was adjusted to the Michaelis–Menten equation and the curve was analyzed with BioDataFit software (V 1.02, Freemont, CA, USA) to obtain the apparent Km value.

### 4.9. [^3^H]-Thymidine Incorporation Assay

Thymidine incorporation during maize germination was performed as described by [32]. Briefly, for each time point, 30 embryos were germinated in 20 mL of MS media supplemented with 100 µL of [methyl-^3^H]-thymidine (1.78 TBq/mmol, 48.0 Ci/mmol; Amersham Pharmacia Biotech, Little Chalfant, UK). The tissue was weighed and ground with 1 mL of homogenization buffer (15 mM NaCl, 100 mM EDTA, 0.5% sodium dodecyl sulfate) and transferred to a test tube. One ml of 20% trichloroacetic acid (TCA) was added to each tube and incubated in ice for 1 h to precipitate DNA. The precipitate was collected in glass fiber filters (Whatman GF/C, 24 mm) and washed with 10% TCA and then 90% ethanol. The dried filters were placed in vials containing 2.5 mL of scintillation solution and cpm were recorded in a Beckman LS 6000IC scintillation counter.

### 4.10. Subcellular Localization

To evaluate the subcellular location from 35S:ZmTK1-GFP fusion and truncated versions, transformation of protoplasts was performed according to [33]. Leaves of 3- to 4-week-old plants from *A. thaliana* (Col-0) were used for protoplast isolation. The transfection was performed with the polyethylene glycol-calcium method. Protoplasts were transfected with the respective constructs. After 18 h, transfected protoplasts were visualized. Mitotracker was used to stain mitochondria. Fluorescence was monitored with the confocal Microscope Olympus FV1000, using 488 nm/505–545 nm (excitation/emission) for GFP and 581 nm/644 nm (excitation/emission) for mitotracker and chloroplast fluorescence.

### 4.11. Stastistical Analysis

All statistical analysis was performed using the Statistix^®^ software (V.4, Analytical Software, Tallahassee, FL, USA).

## 5. Conclusions

In contrast to Arabidopsis and other dicots, the maize genome encodes a single *TK1* gene. Here we report that *ZmTK1* is expressed ubiquitously in various maize tissues, and transcript levels are associated with enzymatic activity, suggesting a relevant role of the pyrimidine salvage during plant development. Moreover, we confirmed that ZmTK1 was driven to both, mitochondria and chloroplast, by a N-terminal signal sequence. This subcellular location suggested that the role of ZmTK1 is more related with the organelle DNA replication and repair as we observed for the *TK1b* gene of *A. thaliana,* which was consistent with the successful complementation with *ZmTK1* of Arabidopsis *tk1a tk1b* double mutant as well as *tk1b* single mutant, and such complementation also restores normal tolerance to genotoxins that damage organellar DNA in *tk1b* mutants.

## Supplementary Materials

The following are available online at https://www.mdpi.com/2223-7747/9/8/930/s1: Figure S1: Amino-acid sequence alignment of ZmTK1, AtTK1a and TK1b, Figure S2: Subcellular localization of the 35S::ZmTK1Δ84-GFP fusion, Figure S3: Expression of AtTK1a partially complements tk1b deficiency, Table S1: Sequences of oligonucleotide primers employed in this study.
