# Supplementary figures and images for: Maize Thymidine Kinase Activity Is Present throughout Plant Development and Its Heterologous Expression Confers Tolerance to an Organellar DNA-Damaging Agent
Source: Plants (Basel). 2020 Jul 23;9(8):930. doi: 10.3390/plants9080930 (PMC7463494; doi:10.3390/plants9080930)

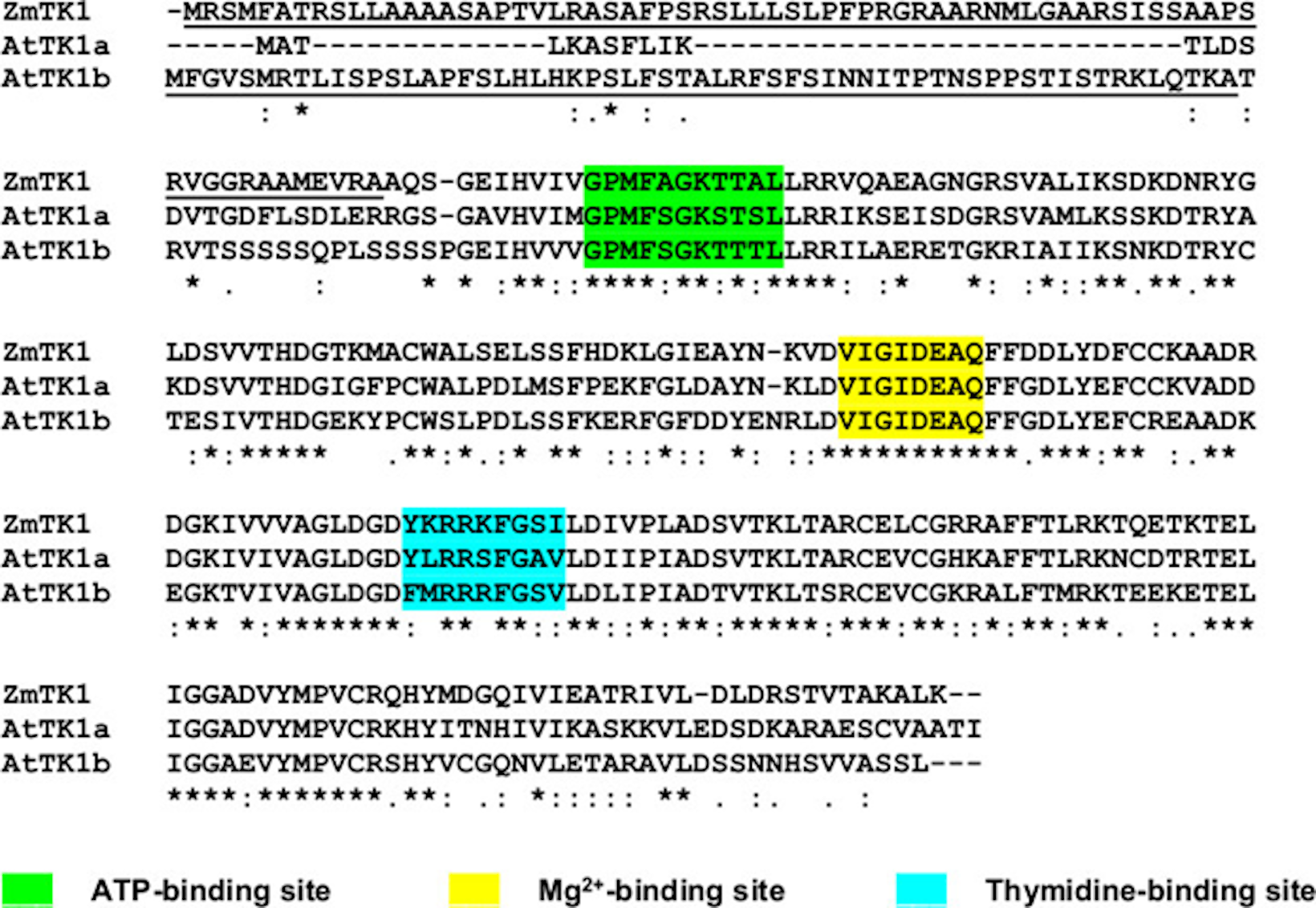

Supplement: Supplementary file 1 [file plants-09-00930-s001.zip › Fig_S1.tif]

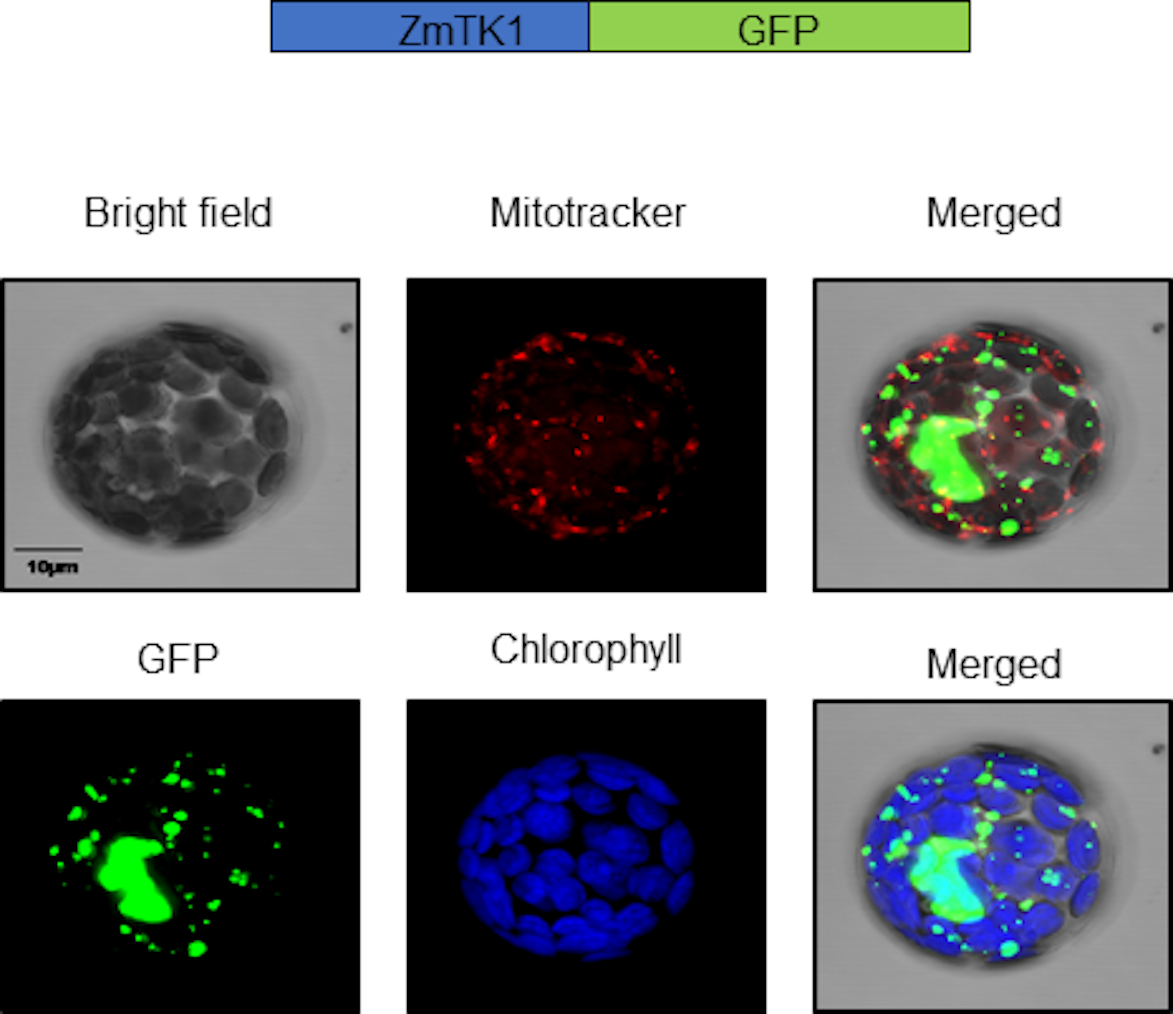

Supplement: Supplementary file 1 [file plants-09-00930-s001.zip › Fig_S2.tif]

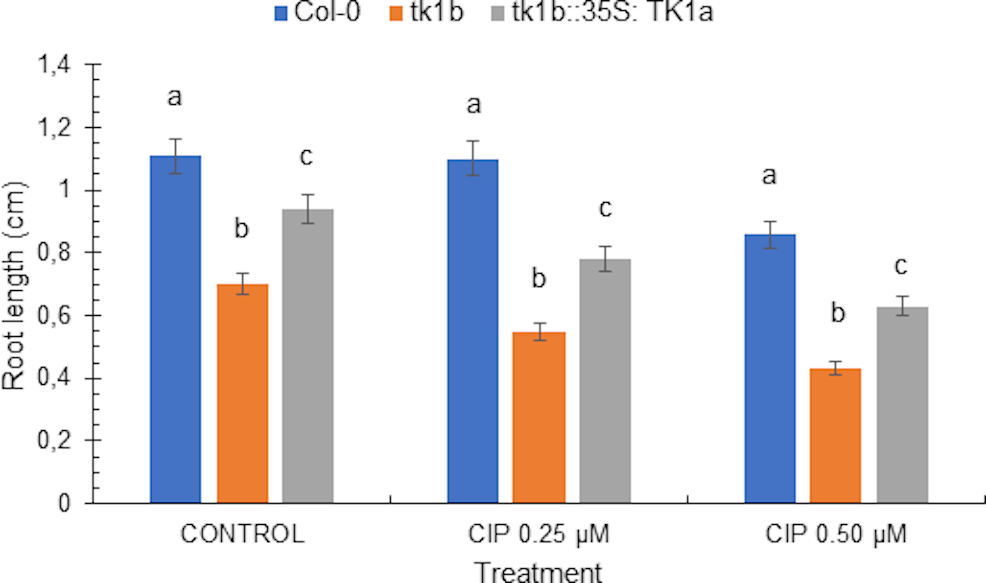

Supplement: Supplementary file 1 [file plants-09-00930-s001.zip › Fig_S3.tif]
